# MicroRNAs as Potential Biomarkers in Cancer: Opportunities and Challenges

**DOI:** 10.1155/2015/125094

**Published:** 2015-03-22

**Authors:** Huiyin Lan, Haiqi Lu, Xian Wang, Hongchuan Jin

**Affiliations:** ^1^Laboratory of Cancer Biology, Institute of Clinical Science, Sir Run Run Shaw Hospital, School of Medicine, Zhejiang University, Hangzhou, Zhejiang 310020, China; ^2^Department of Medical Oncology, Sir Run Run Shaw Hospital, School of Medicine, Zhejiang University, Hangzhou, Zhejiang 310020, China

## Abstract

MicroRNAs (miRNAs) are a group of small noncoding RNAs (ncRNAs) that posttranscriptionally regulate gene expression by targeting their corresponding messenger RNAs (mRNAs). Dysregulated miRNAs have been considered as a new type of ‘‘oncomiRs” or ‘‘tumor suppressors,” playing essential roles in cancer initiation and progression. Using genome-wide detection methods, ubiquitously aberrant expression profiles of miRNAs have been identified in a broad array of human cancers, showing great potential as novel diagnostic and prognostic biomarkers of cancer with high specificity and sensitivity. The detectable miRNAs in tissue, blood, and other body fluids with high stability provide an abundant source for miRNA-based biomarkers in human cancers. Despite the fact that an increasing number of potential miRNA biomarkers have been reported, the transition of miRNAs-based biomarkers from bench to bedside still necessitates addressing several challenges. In this review, we will summarize our current understanding of miRNAs as potential biomarkers in human cancers.

## 1. Introduction

According to NIH (National Institutes of Health), a biomarker is described with a characteristic that it is objectively measured and evaluated as an indicator of normal biological processes, pathogenic processes, or pharmacologic responses to a therapeutic intervention [1]. Useful biomarkers can provide great insights into tumorigenesis and facilitate the development of improved therapies. Cancer is not only one of the biggest global killers but also one of the fastest growing causes of death [2]. Hunting for cancer biomarkers capable of providing diagnostic, prognostic, or therapeutic information has become a necessary but challenging work in cancer research. Currently, some available biomarkers such as PSA (prostate-specific antigen) for prostate cancer, CEA (carcinoembryonic antigen) for colorectal cancer, AFP (a-fetoprotein) for liver cancer, CA 125 for ovarian cancer, and CA 19–9 for pancreatic cancer invariably display an inferior sensitivity and specificity, despite their generally clinical applications. Due to high rates of “overdiagnosis” and “pseudodisease” by PSA test, the USPSTF (US Preventive Services Task Force) issued a grade D recommendation to recommend against routine PSA-based screening for prostate cancer in July 2012 [3]. Therefore, it is an urgent request for more reliable biomarkers to serve as precise indicators of developments and changes of cancer at the cellular levels and the patient's response of cancer therapy.

The discovery of miRNAs has captured increased attention of researchers recently. Following the Lin-4 in* C. elegans*, known as the first member of miRNAs, discovered in 1993 [4], more and more miRNAs have been found and investigated; herein, a number of miRNAs have been identified to essentially revolve in human carcinogenesis by acting as “oncoMirs” or “tumor suppressive miRNAs” [5]. Strikingly, based on the pervasive cancer-associated aberrant expression profiles of dysregulated miRNAs, together with the characteristics of miRNAs such as temporal and spatial specificity, sensitivity, and stability in both paraffin sections and body fluids, miRNAs have shown great potential as a new class of biomarkers in cancer. Furthermore, with the extensive use of next generation of technologies such as miRNA microarrays and high-throughput deep sequencing techniques [6], translating biomarker into practice with increased diagnostic and therapeutic sensitivity and specificity would be less of a problem [7]. Among these contexts, the potential of miRNAs being involved in carcinogenesis as cancer biomarkers has been extensively investigated and developed.

In this review, we will overview recent advances in the dysregulation of tumor-associated miRNAs in both biopsies and circulation and highlight some critical challenges for the transition of miRNAs as cancer biomarkers from a research setting into clinical application.

## 2. Overview of miRNAs: Definition, Biogenesis, and Functions

miRNAs belong to the heterogeneous class of ncRNAs, of 22–24 nt RNAs that play important regulatory roles by targeting mRNAs for cleavage or translational repression [8]. By definition, miRNAs are small RNA molecules incapable of encoding proteins, but possessing important structural, catalytic, and regulatory functions. According to the latest miRBase release (v21, June 2014, http://www.mirbase.org/), there are 28645 entries representing hairpin precursor miRNAs from 223 species, expressing 35828 mature miRNA products. MiRNA genes are located either in independent noncoding DNA loci or in the introns of protein-coding genes [9]. Furthermore, up to 50% miRNAs are clustered on chromosomes with a common promoter [10] and cotranscribed to form different miRNAs families which share common mRNAs targets and biological processes because of their identical seed regions [11].

It is well established that miRNA biogenesis is a complex process which generally includes three main steps as follows. (i) In the nucleus, miRNA genes are transcripted by RNA polymerase II to form primary miRNA transcripts (pri-miRNAs) [12]. (ii) Subsequently, the Drosha RNase III endonuclease trims the pri-miRNA to liberate a pre-miRNA hairpin which is actively transported to the cytoplasm by Ran-GTP and exportin-5 [8, 13]. (iii) Its final maturation is processed in the cytoplasm where Dicer RNase III endonuclease cleaves the pre-miRNA into a single-stranded mature miRNA [13] and then the mature miRNA binds to proteins of the argonaute (Ago) family and assembles the RNA induced silencing complex (RISC) together to exert its further physiological functions.

After being incorporated into the RISC, the mature miRNA induces posttranscriptional gene silencing by tethering RISC to be partially complementary to the target mRNA predominantly found within the 3′-untranslated region (UTR) [14]. Inspiringly, a recent study showed that a single miRNA may repress more than 100 mRNAs on average and over 60% of human protein-coding genes are conserved targets of miRNAs [15]. Given this vast majority of mRNA targets regulated by miRNAs, aberrant miRNA expression profoundly influences a wide variety of cell regulation pathways important to cell proliferation [16], apoptosis [17], and stress responses [18]. In cancer disease phenotype, miRNAs serve exclusively as a novel class of oncogenes or tumor suppressor genes named “oncomiRs” or “tumor suppressive miRs” [19]. Following the first identified tumor suppressive miRNA gene miR-15a/miR-16-1 revolving in the development of B-cell lymphocytic leukemia [20], a growing number of oncomiRs have been validated as well as tumor suppressive miRs (more details are in the review [21]). Remarkably, it has been continually confirmed by numbers of studies that “oncomiRs” or “tumor suppressive miRs” revolve in a multitude of cancer-related signaling pathways. For example, miR-17 activates PI3K/AKT pathway and regulates tumor growth [22], the axis of p53-miRNA-34 network regulates Wnt pathway [23] and promotes EMT program [24], and let-7 leads to growth inhibition by negatively regulating RAS pathway [25]. Taken together, these observations give a miRNAs-mediated complex network model that connects oncogenic and tumor suppressive signaling pathways in human cancers, indicating that miRNAs may play a more indispensable role in the pathogenesis of human cancers than previously thought.

## 3. miRNAs as Potential Biomarkers in Cancer

### 3.1. Aberrant miRNA Expression Profiles in Cancer

#### 3.1.1. Linkage of Aberrant miRNA Expression Profiles with Cancer Development

Aberrant expression of miRNAs in cancer is characterized by abnormal expression levels of mature or precursor miRNA transcripts in comparison with those in the corresponding normal tissues. A steadily growing number of reports have proven that human cancers frequently show an aberrant expression profile of miRNAs as well as pre-miRNAs. With various kinds of high-throughput sequencing platforms applied to analyze the genome-wide expression of miRNA genes these years, aberrant expression profiles of miRNAs either downregulated or upregulated have been discovered in a broad variety of human malignant cancers, including lymphoma [26], breast cancer [27], colorectal cancer [28], prostate cancer [29], and glioma [30].

Although the causes of aberrant miRNAs expression in cancer have not yet been fully elucidated, several defined mechanisms can be possible, including chromosomal abnormalities, genomic mutations, epigenetic changes, and alterations in miRNA biogenesis. Notably, some miRNA genes are frequently located at fragile sites and genomic regions related to cancer [31]. In other words, alterations of miRNAs expression can directly reflect the chromosomal or genomic changes of cancer-associated genes. Together with important roles of cancer-associated miRNAs identified in various types of cancer cell lines [32] and clinical tumor specimens [33–35], the aberrant expressions of miRNAs appear to show significant values of clinical applications.

#### 3.1.2. Diagnostic, Prognostic, and Predictive Values of miRNA Signatures for Cancer

Given those circumstances above, alterations of miRNAs expression in different types of neoplasia constitute specific patterns of miRNA signature for certain types of cancer. Logically, these specific miRNA signatures would be attractively conducive to early diagnosis, signifying the prognosis and predicting the response of cancer therapy.

*(i) Diagnosis and Classification*. It was observed that, with cancers derived from different epithelial and hematopoietic lineage having distinct miRNA profiles, miRNA signatures were able to discriminate them according to their developmental origins. For example, in a research trying to identify cancers with unknown primary tissue of origin by using microarray platform to evaluate the expressions of 47 miRNAs in 101 FFPE (formalin fixed and paraffin embedded) samples from primary or metastatic cancers, overall, the accuracy reached 100% for primary cancers and 78% for metastatic cancers. When the signature was applied to an independent published dataset of 170 samples, correct prediction was further verified in 86% of the metastasis cases [36].

For patients with malignant cancers, precise determination of tumor subtype significantly influences the decision-making of treatments. For example, In renal cell carcinoma (RCC), a research team developed a classification system applying method of decision trees that can distinguish different RCC subtypes in 94 different subtype cases using unique miRNA signatures analysis [37]. This system obtained a sensitivity of 97% for differentiating normal cases from RCC patients. And among the four types of RCC (clear cell RCC (ccRCC), papillary RCC (pRCC), oncocytoma, and chromophobe RCC (chRCC)), 100% for ccRCC subtype from the other fours, 97% for pRCC subtype from oncocytoma and chRCC, and 100% accuracy for distinguishing oncocytoma from chRCC subtype [37]. Moreover, another study by Gilad et al. validated a miRNA-based assay capable of differentiating between four main types of lung cancer in an independent set of 451 samples. They got a result for more than 90% of the samples with overall accuracy of 94%, with a similar performance observed in pathologic and cytologic samples [38]. Collectively, these findings above suggested that miRNAs signatures were able to serve as an accurate classification tool for different cancers in pathologic and cytologic samples.

Moreover, the need of biomarkers for cancer early diagnosis is extremely important because of the fact that survival and prognosis of patients extensively depend on the stage of tumor at the time of detection, with an early diagnosis generally associated with a better prognosis. Promisingly, miRNA signatures have been identified to possess a robust potential as early diagnosis biomarkers. For example, overexpression of miR-205 and miR-21 in ductal adenocarcinoma has been reported to precede phenotypic changes in the ducts [39], suggesting that aberrant miRNA production was an early event in the development of cancer and thus supporting the possibility to use them for early detection of cancers. 

*(ii) Prognostic Values*. In addition to cancer diagnosis including distinguishing tissue of origin, subtypes, and early detection of cancer, miRNA signatures can be also valuable for cancer prognosis prediction, which has been vilified by a number of studies recently. The first evidence was proposed by Calin et al. in chronic lymphocytic leukemia (CLL), where a unique miRNA signature composed of 13 genes can differentiate CLL patients with abnormal expression of prognostic factors (ZAP-70 and IgV(H)) from normal cases. Besides, the same miRNA signature was associated with the presence or absence of disease progression [40]. Moreover, in lung cancer, a signature of five miRNAs has been used in the prediction of treatment outcome for non-small-cell lung carcinoma (NSCLC), among which high expression of miR-221 and let-7a is associated with good prognosis, as opposed to elevated miR-137, miR-372, and miR-182 with poor prognosis [41]. Likewise, high expression of the miR-183 family (miR-183, miR-182, and miR-96) was associated with overall poor survival in patients with lung cancer [42]. In addition, a recent study interrogated plasma miR-10b and miR-373 levels, which are known mediators of metastasis in breast cancer, and they uncovered association of these miRNAs for detecting the lymph node metastasis, thereby showing potential as prognostic biomarkers [43]. It is worth noting that one miRNA alone can also possess accurate predictive power which was clarified by a study in patients with breast cancer, they identified the fact that overexpression of miR-210 is associated with an increased risk of recurrence and a reduced chance of relapse-free survival, and miR-210 alone allowed prediction of prognosis to the same level as a 76-gene mRNA signature test (GENE76) [44]. In prostate cancer, miRNAs have also been connected to prognosis of PC, such as miR-141 and miR-375, which turned out to correlate with high Gleason score or lymph-node positive status in a second independent validation study [45]. Besides, signature of miR-410 and miR-645 was negatively associated with overall survival in advanced serous ovarian cancer [46]. Remarkably, a pattern of seven-miRNA signature recently identified in gastric cancer can predict overall survival and relapse-free survival [47], and, in patients with stage II colon cancer, a six-miRNA-based classifier established was an independent prognostic factor for disease recurrence [48].

*(iii) Predictive Values for Response to Therapy*. Apart from their use as prognostic biomarkers, miRNA signatures could also predict the responses to various cancer managements. The correlation between miRNA signatures and the responses of specific therapies is of great clinical value because of the involvement of miRNAs in chemoresistance identified in many studies [49, 50]. For instance, Meng et al. firstly found that inhibition of miR-21 and miR-200b increased sensitivity to gemcitabine in cholangiocarcinoma cell lines [51]. Subsequently, it was proven that high miR-21 expression involved in gemcitabine chemoresistance was able to predict significantly shorter overall survival (OS) in pancreatic cancer treated with gemcitabine [52]. Likewise, by conferring taxol resistance through the suppression of Bak1 expression, high expression of miR-125b in breast cancer predicted the poor response to taxol-based treatments [53]. In colorectal cancer, the presence of the KRAS mutation was associated with upregulation of miR-127-3p, miR-92a, and miR-486-3p and downregulation of miR-378, which constituted a miRNA signature capable of predicting colorectal cancers resistant to EGFR antagonists [54]. Additionally, it was demonstrated that let-7g and miR-181b may be indicators of chemotherapy response to S-1 based chemotherapy in CRC [55]. In glioma cancer, miR-181d upregulation may correspond to a better response to temozolomide, while upregulation of miR-21 may predict poor response caused by temozolomide resistance [30]. Remarkably, in relapsed patients with NSCLC, the loss of heterozygosity of miR-128b, an EGFR regulator, was correlated with response to the EGFR inhibitor gefitinib [56]. In belief, given these specific miRNA signatures serving as predictors of response to various cancer management, it may be possible to improve patients' prognosis by selecting the right treatment course as soon after diagnosis as possible and permit rapid adaption of treatment to the acquisition of chemotherapeutic and radioresistance. What is more is that, by predicting response to molecular-targeted agents, the use of predictive biomarkers could be promisingly conducive to accelerate the development of new anticancer therapies in clinical trials [57].

In summary, based on the aberrant miRNA expression profiles identified in human cancers, miRNAs give us a totally new horizon in understanding the development management of cancer. Despite of some certain miRNAs commonly exhibiting altered expression across various kinds of tumors, particular types of cancers often express their exclusive patterns of miRNAs referring to their tissues of origin [33]. Such specific miRNA signatures exhibited in types of tumors mentioned above are also termed as “miRNome,” which characterizes the malignant state and defines some of their clinicopathological features such as grade, stage, aggressiveness, vascular invasion, and proliferation index [31]. Given the great value of miRNA signatures for cancer diagnosis, prognosis and prediction of therapeutic responses, a comprehensive list of miRNAs identified as various biomarkers in different cancer types are summarized in Table 1.

### 3.2. Potential Source of miRNA-Based Biomarkers

#### 3.2.1. Stability of miRNAs

In addition to the high specificity and sensitivity of miRNA biomarkers in cancer diagnosis as previously described, miRNAs are characterized by robust stability as well in stored RNA samples, including fixed tissue, blood, and other body fluids, which further supports their potential as biomarkers. Recent studies have systematically investigated the stability of miRNAs even after being subjected to severe conditions that would normally degrade mRNA or other types of larger RNAs. For example, in two similar studies, miRNA expression profiles in FFPE from colorectal cancer (CRC) samples dating back up to 10 year were assessed and compared with fresh frozen CRC samples; a high similarity of miRNAs profiles was observed. Moreover, differing formalin fixation times did not significantly influence miRNAs profiles in FFPE specimens in light of quantitative real-time polymerase chain reaction (qRT-PCR) [58, 59]. For another instance of extracellular miRNAs stability reported by Chen at al., they treated some miRNAs with or without RNase A digestion; then they surprisingly found that half of miRNAs remain intact after 3 hours of exposure to RNase A, but all high molecular weight RNAs set as controls were rapidly degraded. More importantly, after being treated with several harsh conditions including boiling, low/high pH (pH = 1/13), extended storage, or freeze-thaw cycles, miRNAs in sera still maintained potent intrinsic stability as expected [60]. Taken together, these data consistently revealed that miRNAs, particularly serum miRNAs, possess considerable resistance to the enzymatic cleavage by RNase A and various severe conditions, so as to maintain their intrinsic stability for long.

Generally, there are mainly three explanations for miRNAs' stability. Firstly, as a class of small molecules with a short length of only 20–24 nt, miRNAs tend to be less subjected to degradation during FFPE sample processing. Secondly, these small RNAs are taken into encapsulation by cell-derived lipid vesicles such as exosomes [61], microparticles [62], or apoptotic bodies [63]. However, the precise mechanisms of miRNAs' selective packaging remain elusive yet. Thirdly, the nuclease-resistant extracellular miRNAs were predominantly associated with RNA binding proteins such as argonaute proteins (Ago1 or Ago2), nucleophosmin (NPM1), or ribosomal proteins [64], which exert a robust protective effect on miRNAs from abundant nucleases in extracellular space. Overwhelmingly, Turchinovich et al. argued that most of the extracellular miRNAs are not exclusively associated with cell-exported exosomes; instead, a majority of them are floating outside exosomes to bind to Ago2 protein [64]. Nevertheless, no final conclusion has yet been reached on the main mechanism of how to maintain miRNAs' stability. But what we can confirm is that inherent stability of miRNAs in both FFPE and circulation perfectly presents great advantages for them as ideal biomarkers of cancer.

#### 3.2.2. miRNAs in FFPET

Tissue biopsy specimen in the form of FFPET (FFPE tissue) is commonly used for tissue storage, which preserves good tissue integrity so as to provide excellent resources for discovery of disease-detecting biomarkers, not excepting the miRNAs-based biomarkers of cancer certainly. Better than mRNAs which are too unstable to be suitable for qRT-PCR, miRNAs are stable enough to be well preserved even over prolonged FFPE block storage and quantified from FFPET by qRT-PCR. In a word, miRNAs from substantial FFPET can provide tremendous source for cancer-related biomarkers.

Numerous studies have discovered and evaluated a broad variety of potential miRNAs biomarker candidates in FFPET which provide valuable information for cancer diagnosis and prognosis. A recent study used bioinformatics to screen miRNAs classifiers in 69 FFPE pancreatic specimens including benign, premalignant, and malignant. Different miRNA panels mapped to these lesions above, separate anyone from the two others with sensitivity and specificity up to 85%–100% [65]. Furthermore, in another study by Casanova-Salas et al. [66], an independent cohort of 273 FFPET samples was used for validation of new potential biomarkers by qRT-PCR. They assessed the relationship of two miRNAs (miR-182 and miR-187) most differentially expressed in prostate cancer with the clinicopathological characteristics and outcome of patients, and it was observed that miR-187 expression was decreased in advanced prostate cancer cases, which was consistent with microarray data. Moreover, the higher expression of miR-182 independently conferred a worse prognosis for BPFS and PFS using Cox proportional hazard multivariable analysis. Therefore, such results showed the promising potential of miRNAs for serving as biomarkers in cancer diagnosis or prognosis.

In addition, other miRNAs identified as potential biomarkers in FFPET for various types of malignancies are summarized in Table 2.

#### 3.2.3. miRNAs in Blood (Total Blood, Serum, Plasma, and Exosome)

Apart from the diagnostic miRNA signatures identified in specimens from tumor tissues, studies have also shown the diagnostic and prognostic usefulness of miRNAs in circulation. In fact, except for miRNAs in the biopsies and tumor samples, a substantial number of cancer-related miRNAs can be also detected in blood including total blood, serum, and plasma, as well as other body fluids such as saliva, urine, breast milk, seminal fluid, and tears [67].

In fact, the intriguing phenomenon of cancer-associated nucleic acids in circulation has been identified for decades. Generally, from their intracellular origin, miRNAs can be secreted extracellularly in cell-derived extracellular vesicles such as exosomes or bound to other lipoproteins as mentioned above, although the concrete mechanisms for the origin of such miRNAs released by tumor cells have not yet been clarified and little is known about the mechanisms of how to specifically direct miRNAs into multivesicular bodies or exosomes.

However, what we know at present is that miRNAs in blood released by tumor cells may not simply act as a set of bystander product of tumor cells. It has been widely identified that tumor-released (TR) exosomes could act as intercellular mediators transporting diverse types of proteins, mRNA, and miRNAs that can promote angiogenesis, cell proliferation, and cell survival, resulting in establishing an oncogenic niche systemically [68]. Recently, a paradigm of autocrine and paracrine miRNA signaling pathway contributing to microenvironment of cancer through TR exosomes has been proposed. For example, Skog et al. firstly found that the messengers encoding for EGFRvIII protein and miR-21 are transported in glioblastoma-derived exosomes and these molecules could be taken up by normal host cells and transformed into functional signals, stimulating proliferation of cancer cells [69]. Furthermore, Fabbri recently demonstrated that miR-21 and -29a secreted by tumor cells via exosomes could bind to toll-like receptor (TLR) 7/8 and activate these receptors on immune cells, leading to TLR-mediated NF-*κ*B activation and secretion of prometastatic inflammatory cytokines that may ultimately lead to tumor growth and metastasis [70]. These observations above revealed that the mobile miRNAs entrapped in exosomes in blood could function as hormones, entering the circulation and travelling to distant organs to exert their hormone-like effects, leading to widespread consequences within the recipient cells at a distance from the donor cells.

Additionally, Pigati and colleagues verified the fact that the cancer-related miRNAs are specific to the cancer cells, which is a further consideration supporting detection of the circulating miRNAs to serve as molecular tumor markers. They found that the bulk of miR-451 and miR-1246 produced by malignant cells was selectively released into blood particularly, but the majority of these miRNAs produced by normal cells were retained intracellularly [71]. Their findings indicated that release of miRNAs into blood and other biological fluids is selective presumably with an explanation that the assortment of them might be changed by malignant transformation [72].

Therefore, with a stable form and extensively hormone-like effects, the noninvasively detectable cancer-specific miRNAs in circulation have been widely investigated and demonstrated to efficiently discriminate cancer patients from healthy people, predict the prognosis, and monitor the therapeutic response. Comprehensively, recent studies for discovery of potential miRNAs biomarkers for types of cancers in blood and other body fluids have been listed in Table 3.

In serum, following the first paradigm showed that the diagnostic and prognostic potential of miR-21 that was associated with increased relapse-free survival of diffuse large B-cell lymphoma (DLBCL) patients by Lawrie et al. [73] and lots of new candidate miRNA biomarkers in serum of cancer patients have been reported. For example, Chen et al. demonstrated that a profiling of 10 differentially expressed serum miRNAs selected from a sample set including 400 NSCLC cases and 220 controls provided a high sensitivity and specificity for NSCLC diagnosis. They used risk score analysis to evaluate the diagnostic value of the serum miRNAs profile system and found that these 10 miRNAs were able to distinguish NSCLC cases from controls with sensitivity of 93% and specificity of 90%. What is more is that these 10 serum miRNAs were correlated with the stage of NSCLC patients [74]. Moreover, in a recent study, Wu et al. reported 35 miRNAs targeting 11 genes involved in the TGF-*β* signaling pathway for their associations with survival among 141 highly expressed serum miRNAs collected from 391 patients with advanced NSCLC. They concluded that 17 miRNAs were significantly associated with 2-year patient survival using Cox regression model. Of these 17 miRNAs, miR-16 exhibited the most statistical significance, whose high expression was associated with an obviously better survival [75]. Findings of similar crosstalk between TGF-beta signaling and miRNAs machinery also have been clarified in other cancers such as CRC [76] and glioblastoma [77].

In plasma, Mitchell et al. firstly proved that miRNAs are readily measurable by direct cloning and sequencing from a plasma small RNA library. To further determine whether tumor-derived miRNAs enter the circulation at levels sufficient to be measurable as biomarkers, they established a mouse prostate cancer xenograft model system, collected the plasma, and isolated miRNAs for analyzing the different expression of miRNAs compared to the control. They found that levels of miR-629 and miR-660 were able to independently differentiate xenografted mice from controls with 100% sensitivity and 100% specificity [78]. Such conclusion, thereby, provided a firm grounding of the fact that that tumor-derived miRNAs measured in plasma can serve as noninvasion biomarkers for cancer detection. Examples are numerous. Plasma miR-21, miR-145, and miR-155 used in combination helped in distinguishing lung cancer from healthy smokers with 69.4% sensitivity and 78.3% specificity [79]. Combination of miR-148b, miR-409-3p, and miR-801 discriminated powerfully between breast cancer cases and healthy controls [80]. Three plasma microRNAs (miR-106b, miR-20a, and miR-221) had a statistically significant elevation in gastric cancer patients so as to serve as novel biomarkers for the early detection of GC [81]. Interestingly, the combination of miR-16, miR-196a, and CA19-9 was more effective for pancreatic cancer diagnosis, especially in early tumor screening [82]. Ng et al. revealed that expression of miR-92 in plasma is significantly elevated and differentiated CRC from gastric cancer or normal subjects of CRC patients with a high sensitivity of 89% and specificity of 70%; thus miR-92 in plasma could have roles as a potential marker for CRC screening [68]. Moreover, high expression of miR-27b, miR-148a, and miR-326 was associated with decreased progression-free survival of CRC patients [68].

Exosomes are a class of small (50–90 nm) membrane vesicles formed in the plasma membrane by the fusion of endocytic vesicles and extracellularly released into surrounding body fluids [83]. As is mentioned above, miRNAs passively released from normal or tumor-lysed cells may be preferentially secreted and packaged into exosomes that provide a protective effect on the stability of extracellular miRNAs [61]. Excitingly, it was discovered that the exosomal miRNAs content was similar to that in the original tumors [84], thus peaking researchers' interests in the use of exosomal miRNA profiles for diagnostic markers of cancer. For example, Hessvik compared miRNAs in PC-3 cells with the noncancerous prostate cell line by microarray and qRT-PCR analysis. It was found that the exosomal miRNA profile released by PC-3 was highly similar to the profile of corresponding parent cells. In a study of ovarian cancer, researchers found that levels of 8 exosomal miRNAs (miR-21, miR-141, miR-200a, miR-200c, miR-200b, miR-203, miR-205, and miR-214) previously demonstrated as diagnostic markers were similar between cellular and exosomal miRNAs, which were significantly distinct from profiles observed in benign disease [84]. In another study, exosomal miRNAs were profiled from the plasma of prostate cancer patients with or without metastases and a distinct set of 11 miRNAs was present at significantly greater amounts in patients with metastases compared to those without metastases. And the association of miR-141 and miR-375 among these 11 microRNAs was confirmed in plasma exosomes from a separate patient cohort with recurrent or nonrecurrent disease, thus demonstrating that changes of miRNAs concentration present in exosomes could be of great value for early detection and tumor staging diagnosis [85]. Collectively, these results suggested that miRNA profiles of circulating tumor exosomes could potentially be used as surrogate diagnostic markers for biopsy profiles and could also serve as potential diagnostic and prognostic biomarkers of cancer.

#### 3.2.4. miRNAs in Other Body Fluids

Although the majority of studies identified circulating miRNAs in serum and plasma, miRNAs have also been detected in other body fluids such as urine, tears, breast milk, and seminal, amniotic, bronchial, lavage as well as pleural, peritoneal, and cerebrospinal fluids [67, 86]. Recent studies have confirmed the potential use of tumor-specific miRNAs as diagnostic or prognostic markers for cancer in these body fluids. For instance, miR-125a and miR-200a were identified with significantly lower levels in the saliva for oral cancer detection [87]. The RNA ratio of miRNA-126 : miRNA-152 enabled the detection of bladder cancer from urine with high specificity of 82% and sensitivity of 72% [88]. A subset of six miRNAs (miR-187, miR-18a^*^, miR-25, miR-142-3p, miR-140-5p, and miR-204) was capable of correctly classifying bladder urothelial cell carcinoma (UCC) patients with an overall sensitivity of 84.8% and specificity of 86.5% [89]. In combination of miR-205, miR-210 and miR-708 in sputum distinguished lung squamous cell carcinoma patients from normal subjects with 73% sensitivity and 96% specificity thus helping in the early detection of lung squamous cell carcinomas [90]. What is more is that urinary level of cell-free miR-214 could be an independent prognostic parameter for non-muscle-invasive bladder cancer recurrence [91]. Presence of miR-21 and miR-15b in cerebrospinal fluid (CSF) of glioma patients also has been identified as a source of biomarkers [30].

However, as a matter of fact, miRNAs, no matter in serum, plasma, exosomes, or other body fluids, may not be absolutely specific to a certain cancer type. MiR-21 in serum is exactly a representative example for this. From a serial of investigations, it is found that high expression of serum miR-21 in lung cancer associated with poor prognosis [92] and the dynamic change of serum miR-21 between postoperative and preoperative lung carcinoma patients can potentially serve as biomarkers for disease recurrence after surgery operation [93]. In other types of cancer, also, the level of serum miR-21 significantly elevated in patients with CRC and advanced adenoma thus exhibiting its potential diagnostic value for early detection of CRC [94], as well as the prognostic value because of the postoperative reductions in serum miR-21 level which occurred [95]. Observations of analogous diagnostic and prognostic significance also have been identified in esophageal squamous cell carcinomas [96], pancreatic cancer [97], hepatocellular carcinoma [98], and ovarian cancer [99]. Therefore, a concept that has to be noted again is the “miRNome” defined as the full complement of miRNAs in a cell, which emphasizes that different tissues have distinctive patterns of miRNome expression with each tissue presenting a specific signature [100]. In order to map to every type of cancer with higher accuracy and specificity, further studies with larger sample numbers are needed to allow generalized detection of unique patterns of miRNome toward every type of cancer, even before or after therapy, and thus may really help in the identification of useful biomarkers in cancer efficiently.

## 4. Methods for MiRNA Detection

Though miRNAs show striking potential as new biomarkers of human cancers in clinic settings and maintain high stability in both FFPET and body fluids, the methods for miRNAs' detection are still challenging yet for the intrinsic characterisitic of miRNAs such as small size, low level, a high degree of sequence similarity among various members, and tissue-specific or stage-specific expressions. Currently, there are various strategies for the detection of miRNAs, including qRT-PCR, in situ hybridization (ISH), enzymatic luminescence miRNA assay and northern blotting, microarrays, deep sequencing or next-generation sequencing (NGS), and nanopore technology et al. [101]. Herein, qRT-PCR is the most commonly used manner, quantifying the miRNome from low levels with high sensitivity and specificity, which is very important for the lack of large amounts of RNA from clinical samples could be obtained. More importantly, qRT-PCR is more convenient and reliable than methods based on hybridization for its potential as it can be performed in high-throughput assays to allow for large-scale miRNAs detection at the same time and still with high specificity or sensitivity [102]. Therefore, qRT-PCR technique has become the gold standard of nucleic acid quantification currently [103]. Nevertheless, several limitations of qRT-PCR also hinder its use in practice. For example, the selection of inner reference genes is essential and critical for data normalization, but the determination of suitable reference genes as the normalizer remains as a highly controversial issue. As previous studies displayed, the commonly used reference genes include miR-16, miR-142-3p, 18S rRNA, miR-638, let-7a, miR-1249, miR-295, 5SRNA, U6, U6B, RNU38B, RNU43, RNU62, and SNORD43 [104]. Unfortunately, there is no single gene that is constitutively expressed in all cell types and under all experimental conditions [105]. Rigorous experimental justification for these data reported is deficient, and these data are contradicted and inconsistent in their crossing comparison, so none of them has been widely accepted as a standard control. Therefore, further studies are needed to develop a set of stable internal control genes for accurate quantification of miRNAs profiles by qRT-PCR even for each type of human cancers.

Microarray is another technique for miRNAs profiling based on nucleic acid hybridization between target molecules and their corresponding complementary probes. The miRNAs oligonucleotide probes are immobilized to glass slides through covalent crosslinking between the amino groups and the SAM (self-assembling monolayer), forming a ready-to-use miRNA microarray. When hybridized with the miRNA microarray, the isolated miRNAs labeled with fluorescent dye can emit fluorescence after being specifically bound to the corresponding probes. Through detecting the fluorescence signal intensity at different positions on the slides, relative quantities of the corresponding miRNAs can be evaluated consequently [106]. Furthermore, microarray-based techniques are particularly attractive for their powerful high-throughput function allowing for detecting the presence of a wide range of defined miRNAs simultaneously within numerous samples processed in parallel in a single experiment. However, deficiencies of microarray also exist, for example, background noise and crosshybridization problems as well as measuring only the relative abundances of previously discovered miRNAs [107].

Deep sequencing relying on next-generation sequencing machines appears to be another promising quantitative technology for miRNAs profiling, because it provides high-throughput sequencing enabling processing up to millions of sequence reads in parallel simultaneous with high speed [108]. In this method, adapters are ligated to the prepared cDNA libraries from RNA sample of interest, followed by the massively parallel sequencing of millions of individual cDNA molecules from the library. Bioinformatic analysis translates the sequence reads into miRNAs abundance levels with relative quantification and then discovers novel miRNAs differentially expressed and their associated genes by using digital approaches may be publicly available or custom-made software tools [107]. Compared with the microarray, deep sequencing technologies are not subject to the problems of background noise and crosshybridization; they measure absolute abundance over a wider dynamic range than microarrays and are not limited by array content, allowing for reflecting the actual picture of the miRNA profiles and the discoveries of novel miRNAs having not been previously identified [109].

Nanopore single-molecule technology, a novel method recently reported by several studies to electrically detect miRNAs in tissue or biofluids, requires no labeling, enzyme reactions, or amplification [110, 111]. Wang et al. showed that a nanopore sensor, which generates a target-specific signature signal by using a programmable oligonucleotide probe, were capable of quantifying subpicomolar levels of cancer-associated miRNAs and distinguishing single-nucleotide differences between miRNA family members [111]. Coinciding with the work of Wang et al., another study by Wanunu et al. used a 3 nm synthetic nanopore to detect the complex of liver miR-122a hybridized with the probe: miRNA duplex enriched through binding to the viral protein p19, and then the abundance of the duplex was quantified using a nanopore, providing accurate quantification for cellular miRNAs in tissues finally [110]. Taken together, these works by them demonstrated that the nanopore-based miRNAs techniques may with promising potential for quantitative miRNAs detection to discover more novel biomarkers and thus it would be validated as noninvasive method for early diagnosis in cancer patients.

## 5. Perspectives and Challenges

Years of research work have indicated that miRNAs play important regulatory roles in translational repression, eventually contributing to the cancer development. Indeed, gene profiling studies have identified a number of significant dysregulated miRNAs as “oncomiRs” or “tumor suppressors” in a variety of human cancers, and overwhelming amounts of data have strongly validated that the ubiquitously aberrant expressions of miRNAs were closely associated with the pathogenesis and progression of human malignancies. These dysregulated miRNAs constitute unique patterns of miRNome in different categories of cancers, which provide accurate fingerprints to detect and classify various cancers. Particularly, as circulating miRNAs in blood and other body fluids are readily detected by various techniques in a relatively noninvasive manner, the clinical application of miRNAs as a new generation of cancer biomarker is considered to be prospecting.

Despite the encouraging results, we are still confronted with many difficulties on the long way of transiting miRNAs as biomarker from bench to bedside. First, exactly as the proposing of the misconceptions that the 98% in human genome is noncoding and thus junk DNA, miRNAs are probably only the tip of the iceberg of noncoding genes in our complex genome. More miRNA transcripts remain to be excavated extensively. Second, more fundamental investigations are needed to further elucidate the exact miRNAs' functional roles in cancer biology. Apart from the pathway by targeting mRNAs for cleavage or translational repression, if there are any other unrevealed mechanisms led to the translational repression, or even any other undiscovered regulation functions more than posttranscriptional regulation. Third, it is needed to further clarify the potential biological functions of circulating miRNAs, particularly the mechanisms of MVBs-contained miRNAs for cell-cell communication, tumor immune evasion, and tumor microenvironment. After all, the underlying mechanism of miRNAs secretion or the mechanisms of the selectivity of miRNAs packaging are still elusive. Fourth, more consistent and reliable miRNA signatures or miRNome in both FFPET and circulation are urgent to be established by adequately large sample size of cohort studies on multiple, independent populations. Fifth, methods for the detection and analysis of miRNAs should be further optimized. To ensure the miRNAs' detection results are as consistent as possible, a set of technical methods both miRNA extraction procedures and technology platforms should be compared with each other or optimized for different types of cancer patients. In addition, it is worth noting that further studies are needed to develop a set of stable inner reference genes to accurately quantify miRNAs even for each type of human cancers.

Despite such challenges that are a lot, the potential of miRNAs as a new class of cancer biomarkers is attractive and cannot be underestimated. MiRNAs are being expected to bring an exciting new dimension to the field of clinical management for human cancers in the near future.

## Figures and Tables

**Table 1:** Types of specific miRNAs biomarkers in different cancers.

Cancer	Type of biomarker	miRNAs biomarkers	Reference
Breast cancer	Diagnostic	miR-10b, -21, -30a, -92a, -125b, -141, -145, -200, -801, -155, -191, -203, -210	[112, 113]
	Prognostic	miR-10b, -373, miR-210	[43, 44]
	Predictive	miR-125b	[53]

Lung cancer	Diagnostic	miR-21, -155, miR-16, miR-17, -19b, -25, -29c, -30c, -106a, -126, -451, -660, -28-3p	[114–116]
	Prognostic	miR-221, let-7a, -137, miR-372, -182	[41, 42]
		miR-1, -15b, -16, -21, -126, -142-3p, -148a, -197	[116]
	Predictive	miR-128b	[56]

Liver cancer	Diagnostic	miR-222, -223, -181a, -181b, -181c, -200c, -203, -21, -224, -10b, -222, 126, -96	[117, 118]
	Prognostic	miR-21, -22, -26, -29, -31, -122, -124, -135a, -139, -145, -146a, -155, -200c, -221, -222, -222, -223	[119]
	Predictive	miR-21, -200b	[52]

Colon cancer	Diagnostic	miR-15b, -18a, -29a, -335, miR-17-3p, -20a, -21, -92, -601, -760, -29a	[120, 121]
	Prognostic	miR-141	[122]
	Predictive	miR-127-3p, -92a, -486-3p, -378, let-7g, miR-181b	[54, 55]

Pancreatic cancer	Diagnostic	miR-205, -21, miR-642b, -885-5p, -22	[39, 123]
		miR-145, -150, -223, -636, -26b, -34a, -122, -126^*^, -145, -150, -223, -505, -636, -885.5p	[124]
	Prognostic	miR-130b, miR-21, -105, -196a-2, -203, -210, -222, -452, -105, -127, -187, - 518a-2, - 30a-3p	[125, 126]
	Predictive	miR-21	[52]

Prostate cancer	Diagnostic	miR-30c, -622, -1285, miR-10b, -373, let-7c, -7e	[43, 127]
		miR-141, -375, miR-26a, -195	[128]
	Prognostic	miR-141, -375, miR-20a, -21, -141, -145, -221	[45, 129]
	Predictive	miR-21	[130]

Ovarian cancer	Diagnostic	miR-200 family, let-7 family, miR-21, -29a, -92, -93, miR-126, -127, -132, -155, -214, -182, -205, -144, -222, -302	[131–133]
	Prognostic	miR-410, -645, miR-200 family, miR-141, -429	[46, 134]
	Predictive	miR-23a, -27a, -30c, let-7g, -199a-3p, -181a, -181b, -213	[132]

**Table 2:** miRNAs as potential biomarkers in FFPE samples.

Cancer	miRNAs as biomarkers	Expression	Reference
Glioblastoma	miR-323, -329, -155, -210	Up	[135]
	miR-326, -130a	Down	[135]
	miR-21, -221	Up	[136]
	miR-128, -181b	Down	[136]

Head and neck cancer	miR-21, -31, -107, -138, -504, -10b	Up	[137]
	miR-16, -20a, -106b, -142-3p	Up	[138]
	miR-155, -423, -451, let-7i	Up	[138]
	miR-10a, -125b, -375	Down	[138]

Breast cancer	miR-21, -29b-2, -125b	Up	[139]
	miR-16, -155, -191, -196a	Up	[139]
	miR-34b, -145, -205, let-7a	Down	[140, 141]

Lung cancer	miR-21, -205	Up	[142]
	miR-16	Up	[115]

Liver cancer	miR-519d	Up	[143]
	miR-146a	Down	[144]
	miR-185	Down	[145]

Gastric cancer	miR-21, -221, -222	Up	[146]
	miR-10b, -21, -106a, -223, -338	Up	[47]
	miR-30a-5p, -31, -126, let-7a	Down	[47]

Pancreatic cancer	miR-192-5p, -202-3p, -337-5p, -130-3p	Up	[65]
	miR-187, -30a-3p	Up	[147]
	miR-21, -155, -203, -210, -222, let-7a	Up	[148]
	miR-200c	Down	[149]

Cervical cancer	miR-21, -27a, -34a, -146a	Up	[150]
	miR-155, -196a, -203, -221	Up	[150]

Ovarian cancer	miR-223	Up	[151]
	miR-9, -200a, -200b, -429	Down	[151]

Prostate cancer	miR-31, -143, -221	Up	[152]
	miR-126, -146a, -150	Down	[152]
	miR-182	Up	[66]
	miR-187	Down	[66]
	miR-203	Down	[153]

Colorectal cancer	miR-31, -135b	Up	[154]
	miR-21, -23a, -193a-3p, -338-5p	Down	[155]

Bladder	miR-10a-5p, -31-5p, -130a, -3p	Up	[156]
	miR-145, -30a-3p, -125b, -133a	Down	[157]
	miR-133b, -195, -199a	Down	[157]

**Table 3:** Potential miRNAs biomarkers for types of cancers in blood and other body fluids.

Cancer	Samples	miRNAs biomarkers	Expression	Reference
Glioblastoma	Serum	miR-15b, -23a, -133a, -150, -197, -497	Up	[158]
	Plasma	miR-128, -342-3p	Down	[30]
	CSF	miR-21, -17-5p, -200	Up	[30]

Head and neck	Plasma	miR-31, -10b, -24, -181, -184	Up	[87]
	Saliva	miR-31	Up	[87]
		miR-200a, -125a	Down	[87]

Breast cancer	Serum	miR-373, -155	Up	[159]
		miR-34a, -17	Down	[159]
		miR-222, -103, -23a, -29a, -23b, -24, -25	Up	[160]
	Plasma	miR-148b, -376c, -409-3p, -801	Up	[80]

Lung cancer	Serum	miR-21-3p, -205-5p, -205-3p, -141, -200c	Up	[92]
		miR-21, -24, -205, -30d	Up	[93]
	Plasma	miR-21, -155	Up	[114]
		miR-145	Down	[114]
	Exosome	miR-17-3p, -21, -106a, -146, -155, -191,-192, -203, -205, -210, -212, -214	Up	[161]
	Sputum	miR-205, -210, -708	Up	[90]

Liver cancer	Serum	miR-122	Up	[162]
		miR-500	Up	[163]
		miR-21, -122, -223	Up	[164]
		miR-21, -1, -25, -92a, -206, -375, let-7f	Up	[165]
		miR-16	Down	[166]

Gastric cancer	Serum	miR-221, -376c, -744	Up	[167]
		miR-1, -20a, -27a, -34, -423-5p	Up	[168]
	Plasma	miR-106b, -20a, -221	Up	[81]
		miR-21, -223, -218	Up	[169]
		miR-451, -486	Down	[170]

Pancreatic cancer	Serum	miR-21, -100, -155	Up	[171]
		miR-203, -369-5p, -376a, -375	Up	[172]
	Plasma	miR-21, -210, -155, -196a	Up	[82, 173]
	Exosome	miR-21, -17-5p	Up	[174]

Colorectal cancer	Serum	miR-29a	Up	[175]
	Plasma	miR-17-3p, -92	Up	[68]
		miR-92a, -29a	Up	[176]
		miR-27b, -148a, -326	Up	[177]
		miR-221	Up	[178]
		miR-15b, -17	Up	[179]

Ovarian cancer	Serum	miR-21, -92, -93, -126, -29a	Up	[131]
		miR-155, -127, -99b	Down	[131]
	Plasma	miR-205	Up	[180]
		let-7f	Down	[180]
	Exosome	miR-21, -141, -200a/b/c, -203, -205, -214	Up	[84]

Prostate cancer	Serum	miR-26a-1, -141, -375	Up	[181]
		miR-16, -195, -26a, let7i	Up	[128]
		miR-20b, -874, -1274a, -1207-5p, -93, -106a	Up	[182]
		miR-223, -26b, -30c, -24	Down	[182]
	Exosome	miR-107, -574-3p	Up	[85]

Bladder	Blood	miR-129, -133b	Up	[183]
		miR-26b-5p, -144-5p, -374-5p	Up	[184]
		miR-378g, -942, -106a-5p, -142-3p, -374a	Up	[185]
	Urine	miR-18a, -25, -187	Up	[89]
		miR-142-3p, -140-5p, -204	Down	[89]
		miR-214	Up	[91]
		miR-200, -155, -192, -205	Up	[186]

Large B-cell lymphoma	Serum	miR-21, -155, -210	Up	[73]
		miR-15a, -16-1, -29c, -155	Up	[187]
		miR-34a	Down	[187]

Leukemia	Serum	miR-181b-5p, -10a-5p, -93-5p	Up	[188]
		miR-129-5p, -155-5p, -320d	Up	[188]
		miR-29c, -223	Down	[189]
